# The Effect of Vitamin D Supplementation on Thyroid Hormone Levels in Patients With Autoimmune Thyroid Disease: A Systematic Review

**DOI:** 10.7759/cureus.66062

**Published:** 2024-08-03

**Authors:** Sabaa I Saad-Omer, Shivani Singh, Oluwatoba T Olayinka, Jaslin Orelus, Mah Rukh Nisar, Rudrani Kotha, Naiela E Almansouri

**Affiliations:** 1 Internal Medicine, California Institute of Behavioral Neurosciences & Psychology, California, USA; 2 Clinical Sciences, California Institute of Behavioral Neurosciences & Psychology, California, USA; 3 Emergency Medicine, California Institute of Behavioral Neurosciences & Psychology, California, USA; 4 Medicine, Neurology, California Institute of Behavioral Neurosciences & Psychology, California, USA; 5 Internal Medicine, University of Tripoli, Tripoli, LBY

**Keywords:** hyperthyroidism, hypothyroidism, supplementation, autoimmune thyroid disease, vitamin d deficiency

## Abstract

Autoimmune thyroid diseases (AITDs) pose significant challenges in clinical practice, representing one of the most common endocrine abnormalities. Vitamin D deficiency has been linked as one of the contributing factors to the etiology of AITDs. This systematic review evaluates the effects of vitamin D supplementation on thyroid-stimulating hormone (TSH), triiodothyronine (T3), and thyroxine (T4) levels in adults with AITDs. Using a PICO (population, intervention, comparison, and outcome) framework and adhering to the Preferred Reporting Items for Systematic Reviews and Meta-Analyses (PRISMA) guidelines, seven relevant studies were identified from an initial pool of 1,469 articles. The population comprised individuals with thyroid autoimmunity, as evidenced by at least one elevated positive thyroid autoimmune marker and intervention involved the supplementation of vitamin D, regardless of the dose or method of administration. All randomized clinical trials within the last 10 years, which fit the study criteria, were included. These studies showed varying results based on follow-up duration. Short-term studies (three months or less) demonstrated no significant changes in mean TSH, T3, or T4 levels compared to the control group with vitamin D supplementation. However, all of the long-term studies (greater than three months) indicated significant improvements compared to the control in mean TSH, T3, and T4 levels. Additionally, all long-term studies that compared TSH, T3, and T4 to baseline levels revealed significant changes by the trial's end. Despite these promising findings, the review highlights limitations, including small sample sizes, short study durations, and the need for further research to establish optimal dosing and treatment duration for vitamin D in AITD management. The overall findings suggest that vitamin D supplementation may play a part in thyroid hormone regulation in AITD, particularly with prolonged administration.

## Introduction and background

In clinical practice, thyroid disorders, especially autoimmune thyroid diseases (AITDs), are almost often one of the most predominant endocrine abnormalities, and they are the most common pathological condition of the thyroid gland [1]. It has been proposed that a variety of factors, including genetic, environmental, and hormonal ones such as vitamin D deficiency, contribute to the etiology of AITDs [2]. In a study conducted by Agmon-Levin et al., it was discovered that vitamin D levels were lower in individuals with systemic and organ-specific autoimmune illnesses than in healthy people and that vitamin D supplementation improved autoimmune diseases [3].

There are multiple heterogeneous clinical manifestations associated with AITDs, the main two are Graves disease (GD), if the patient has a predominance of hyperthyroidism and thyroid-stimulating hormone receptor antibodies (TRAb) and Hashimoto thyroiditis (HD), if the patient has a predominance of hypothyroidism and positive thyroid peroxidase antibody (anti-TPO) or positive thyroglobulin antibodies (anti-Tg) [4]. According to a cross-sectional observational study in the Thai population, patients who tested positive for these antibodies were more likely to have vitamin D deficiency and insufficiency than those who tested negative for these antibodies [5]. The presence of lymphocyte infiltrates within the gland is a typical pathogenic characteristic of AITD, regardless of how it presents. Histologically, lymphocytic infiltration, primarily constituted of T-cells, which may gradually replace thyroid follicles, is the hallmark of AITDs [6].

Hashimoto thyroiditis and GD have varying treatment guidelines. The main treatment for Hashimoto's thyroiditis is thyroid replacement therapy; although Vitamin D is recognized, it is currently not listed as a method of treatment by the American Association of Clinical Endocrinologists. [7]. GD, on the other hand, is managed with antithyroid drugs, radioactive iodine, or surgery, and vitamin D currently has no role in the direct treatment plan [8]. This information is a result of the lack of interventional and causality studies demonstrating the effectiveness of vitamin D supplementation in the management of thyroid disorders.

Due to the paucity of research examining the impact of vitamin D supplementation on thyroid hormone levels, the aim of this study was to systematically assess how vitamin D administration affected thyroid function tests, namely, thyroid-stimulating hormone (TSH), triiodothyronine (T3) and thyroxine (T4) in patients with AITDs.

## Review

Method

Search Sources and Search Strategy

In this systematic review, a PICO (population, intervention, comparison, and outcome) framework was developed to offer a structured framework that would guide the methods section [9]. The patient population (P) comprises all adult patients with autoimmune thyroid diseases. The intervention (I) comprises the administration of vitamin D, regardless of the dose and form. Comparison is to the placebo group and/or baseline levels, according to the study (C). The primary outcome (O) to be evaluated is the presence of a significant effect of supplementation on thyroid hormone levels, compared to placebo or baseline levels. 

The Preferred Reporting Items for Systematic Reviews and Meta-Analyses (PRISMA) checklist was followed to provide guidance for this literature review [10]. An online search was conducted using PubMed, Medline, PubMed Central, Cochrane Library, and ScienceDirect. The following combination of search phrases used for all databases included "Thyroid" OR "Hashimoto disease" OR "Graves disease" OR "Thyroid autoimmunity" AND "Vitamin D" OR "Cholecalciferol" OR “25-hydroxyvitamin D” OR “25(OH)D”. This enabled us to narrow down our search, ensuring that only relevant articles were included in this study.

Study Selection and Eligibility Criteria

Articles found following the database search were reviewed to eliminate any duplicates. Initially, the articles were screened carefully by reviewing the title and abstract. Irrelevant articles were removed, and the remaining articles were assessed by reading the full text. Subsequently, any articles not meeting the eligibility criteria were removed.

Inclusion and Exclusion Criteria

All databases were filtered so that only full English articles and studies conducted in the last 10 years appeared. Studies chosen following the screening were subject to removal if they had not satisfied the inclusion criteria or had satisfied the exclusion criteria, as shown in Table 1. The following eligibility criteria must be met: (1) design: randomized clinical trial; (2) population: patients with thyroid autoimmunity, demonstrated by the presence of at least one positive thyroid autoimmune marker; (3) intervention: vitamin D, regardless of the dose and method of administration; and (4) outcome variables: at least one of either TSH, T3, or T4.

**Table 1:** The inclusion and exclusion criteria

Inclusion Criteria	Exclusion Criteria
Papers focusing on adults	Papers focusing solely on children
Studies written in the English language	Studies not written in the English language
Randomized clinical trials	Reviews, editorials, abstracts, unpublished literature, commentaries
Papers with free full access	Papers without free full access
Studies conducted in the last 10 years	Studies conducted before the last 10 years

Risk of Bias and Quality Assessment

The Cochrane Risk-of-Bias (RoB) tool for randomized trials was used to determine the presence of potential bias that may have an effect on the overall quality of this study [11]. The tool evaluates bias in five domains including bias arising from the randomization process, bias due to deviations from intended interventions, bias due to missing outcome data, bias in measurement of the outcome, and bias in selection of the reported result.

Results

**Figure 1:**
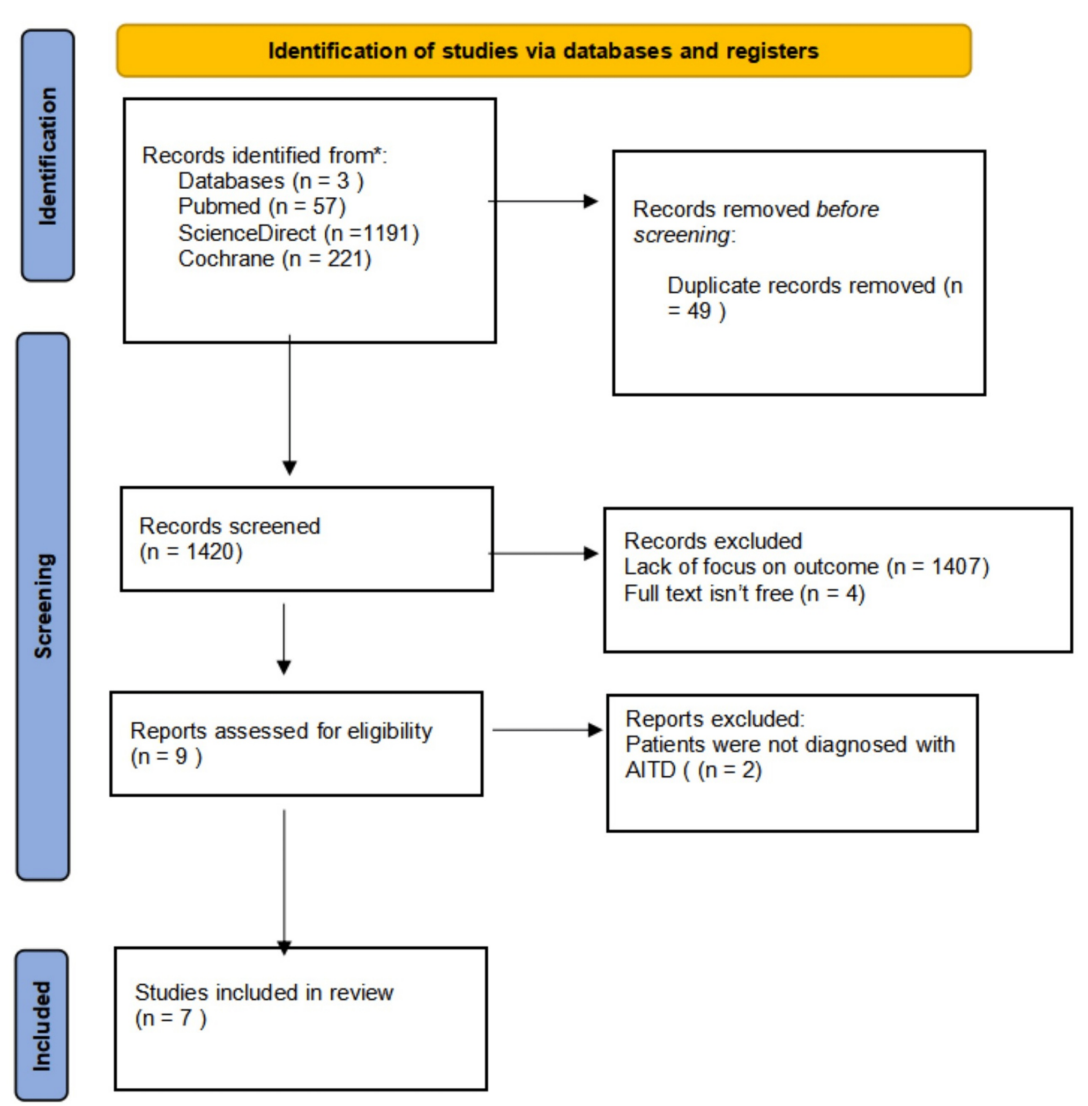
PRISMA flowchart AITD: Autoimmune thyroid disease, PRISMA: Preferred Reporting Items for Systematic Reviews and Meta-Analyses, n: number

Our initial search yielded a total of 1,469 results. These results were obtained from the databases following filters for English language and studies conducted in the last 10 years. The results were uploaded into EndNote, and duplicate articles were removed using the program’s duplicate removal feature. After screening the remaining 1,420 articles according to title, abstract, and inclusion and exclusion criteria, we were left with nine articles, as shown in Figure 1.

**Figure 2:**
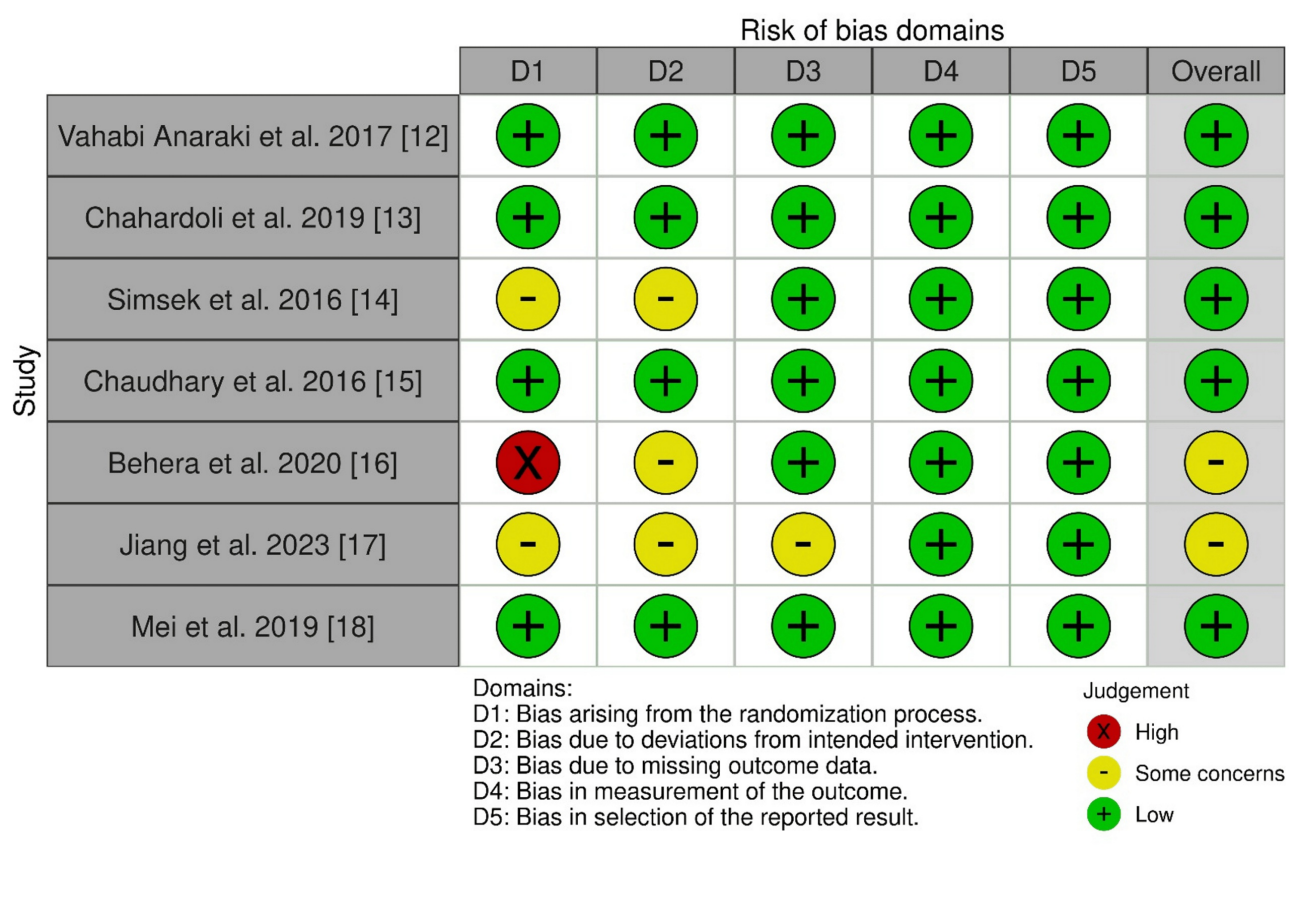
Version 2 of the Cochrane Risk-of-Bias tool for randomized trials Sources: Refs. [12-18]; D: domain

Two of the articles measured autoimmune thyroid markers; however, the patients were not diagnosed with the disease, and therefore the articles were excluded from review. The Cochrane RoB quality assessment was used to check potential bias in the seven remaining studies [11]. Only studies that had low risk or some concerns of bias were included, as shown in Figure 2.

This review involved both adult male and female participants who had been diagnosed with AITD, using a range of thyroid hormones and imaging findings. A summary of the findings of all articles included is provided in Table 2. Three of these studies had a pre-requisite of vitamin D deficiency in selected participants. Different dosages of vitamin D were used; however, the majority of the studies had administered oral vitamin D once per week.

**Table 2:** Study characteristics IU: International units, TPO-Ab: Thyroid peroxidase antibody, TgAb: Thyroglobulin antibody, TRAb: Thyroid-stimulating hormone receptor antibodies

Study	Number of Participants	Establishment of Thyroid Autoimmunity	Presence of Control Group	Pre-existing Vitamin D Deficiency	Vitamin D Form and Dose	Follow-up Period
Vahabi Anararaki et al. 2017 [12]	56	Positive TPO-Ab	Present	Present	oral 50,000 IU weekly	3 months
Chahardoli et al. 2019 [13]	40	Positive TPO-Ab	Present	Present	oral 50,000 IU weekly	3 months
Simsek et al. 2016 [14]	82	Positive TPO-Ab and/or TgAb	Present	Present	oral 1000 IU daily	1 month
Chaudhary et al. 2016 [15]	100	Positive TPO‑Ab	Present	Absent	oral 60,000 IU weekly	8 weeks
Behera et al. 2020 [16]	23	Positive TPO-Ab	Absent	Absent	oral 60,000 IU weekly	6 months
Jiang et al. 2023 [17]	179	Positive TPO-Ab and/or TgAb	Present	Absent	oral 800 IU daily	6 months
Mei et al. 2021 [18]	36	Positive TRAb	Present	Absent	oral 800-1200 IU daily	12 months

Baseline characteristics of study participants across all seven studies is provided in Table 3. No statistically significant differences were found in baseline characteristics in intervention and control groups in all seven studies. TSH, T3, and T4 were also compared at the end of the trial between intervention and control groups to denote whether there were any statistically significant differences. Continuous data were expressed as mean +/- SD or mean (SE), and statistical significance across all studies is denoted as P-value < 0.05. The T3 levels showed significant changes in two of the studies, on the other hand thyroxine (T4) showed significant changes in four of the studies. Notably, all of these studies were conducted in Asia: three in Iran, two in India, two in China, and one in Turkey. All studies demonstrated low baseline levels of mean vitamin D, five of the included studies had overt deficiency [12,14-17], while two demonstrated vitamin D insufficiency [13,18], according to the Endocrine Society [19].

**Table 3:** Baseline characteristics of the study participants Data are expressed as mean ± SD or mean (SE). p-value < 0.05 denotes statistical significance M:F: Male:Female, BMI: Body mass index, TSH: Thyroid stimulating hormone, T3: triiodothyronnine, T4: thyroxine, N/A: Not available

Study	Age (years)			Sex Ratio M:F			BMI (kg/m²)			Baseline Vitamin D (ng/ml)			Baseline TSH (mIU/L)			Baseline T3			Baseline T4		
	Intervention	Control	P-value	Intervention	Control	P-value	Intervention	Control	P-value	Intervention	Control	P-value	Intervention	Control	P-value	Intervention	Control	P-value	Intervention	Control	P-value
Vahabi Anararaki et al. 2017 [12]	43.55	44.12	0.81	0:21	0:15	0.31	27.48	26.9	0.54	13.28 (0.86)	12.76 (0.74)	0.98	3.30 (0.5)	3.45 (0.43)	0.57	N/A	N/A	N/A	N/A	N/A	N/A
Chahardoli et al. 2019 [13]	36±5.2	35.9±7.8	0.81	0:19	0:21	N/A	25.65±5.1	27.80±5.1	0.22	25.3±11.01	19.8±8.8	0.084	3±2.09	2.56±1.36	0.75	1.28±0.34 (ng/ml)	1.32±0.37 (ng/ml)	0.72	11.35±1.82 (μg/dl)	12.1±1.99 (μg/dl)	0.18
Simsek et al. 2016 [14]	35.8±12.0	39.7±12.6	0.171	9:37	5:31	0.702	N/A	N/A	N/A	11.5±5.9	8.6±4.2	0.052	4.1±4.0	4.0±2.5	0.290	N/A	N/A	N/A	1.5±1.1 (ng/dL)	1.1±0.4 (ng/ml)	0.050
Chaudhary et al. 2016 [15]	28.48±6.57	27.86±7.29	0.656	11:59	13:37	0.640	24.03±3.7	23.42±2.94	0.371	33.25 (93.77) nmol/L	39.61 (116.31) nmol/L	0.391	6.88 (138.98)	6.8 (149.46)	0.783	N/A	N/A	N/A	13.90±3.86 (pmol/L)	14.03±3.99 (pmol/L)	0.866
Behera et al. 2020 [16]	35.5±11.03	N/A	N/A	1:22	N/A	N/A	25.2±7.7	N/A	N/A	15.33±5.71	N/A	N/A	7.23±3.16	N/A	N/A	N/A	N/A	N/A	0.9±0.29 (ng/dL)	N/A	N/A
Jiang et al. 2023 [17]	34.86±12.0	33.30± 10.20	0.356	N/A	N/A	N/A	N/A	N/A	N/A	15.39±4.29	15.48±4.41	0.894	3.41±1.57	3.37±1.71	0.873	4.64±0.72 (pmol/L)	4.66±0.82 (pmol/L)	0.849	16.08± 2.79 (pmol/L)	15.91±2.50 (pmol/L)	0.665
Mei et al. 2021 [18]	56.00±6.02	56.78 ±5.91	0.786	7:11	8:10	0.786	N/A	N/A	N/A	27.78±9.96	36.11±9.03	N/A	0.038±0.031 (IU/L)	0.039±0.032 (IU/L)	N/A	18.13±2.38 (pmol/L)	16.52±2.96 (pmol/L)	N/A	54.89±8.24 (pmol/L)	47.61±8.08 (pmol/L)	N/A

Baseline TSH levels were compared at the start of the trial and end of the trial, as shown in Table 4. Only three of the seven studies showed statistically significant changes in the TSH levels. The study by Behera et al. demonstrated a significant decrease in TSH at the end of the trial as compared to baseline levels [16]. The studies by Jiang et al. and Mei et al. demonstrated significant changes in TSH at the trial end as compared to placebo [17,18]. Additionally, the study by Jiang et al. demonstrated a significant decrease in TSH levels at the end of the trial as compared to baseline levels [18].

**Table 4:** Comparison of TSH levels (mIU/L) before and after vitamin D administration in intervention and control groups Data are expressed as mean ± SD or mean (SE). p<0.05 indicates statistical significant difference in intervention group compared to control group after trial end. TSH: Thyroid-stimulating hormone. *statistically significant decrease as compared to baseline in intervention group.

Study		Intervention	Control	P value
Vahabi Anararaki et al. 2017 [12]	Baseline	3.30 (0.5)	3.45 (0.43)	0.16
	After Trial	3.88 (0.82)	2.66 (0.38)	
Chahardoli et al. 2019 [13]	Baseline	3±2.09	2.56±1.36	0.47
	After Trial	1.83±1.4	2.77±1.9	
Simsek et al. 2016 [14]	Baseline	4.1±4.0	4.0±2.5	0.265
	After Trial	3.5±2.5	3.5±2.2	
Chaudhary et al. 2016 [15]	Baselinea	6.88 (138.98)	6.8 (149.46)	0.605
	After Trial	3.16±2.06	3.39±2.19	
Behera et al. 2020 [16]	Baseline	7.23±3.16	N/A	0.001*
	After Trial	3.04±2.62	N/A	
Jiang et al. 2023 [17]	Baseline	3.41±1.57	3.37±1.71	0.000
	After Trial	2.25±1.22	3.58±1.78	
Mei et al. 2021 [18]	Baseline (IU/L)	0.038±0.031	0.039±0.032	<0.001
	After Trial	1.47±0.78	1.49±1.12	

Only three studies explored the changes in mean T3 levels following vitamin D supplementation, as shown in Table 5. The study by Chahardoli et al. demonstrated no statistically significant changes in mean T3 levels at trial end compared to control group [13]. The other two studies demonstrated a significant change in T3 following vitamin D administration [17,18].

**Table 5:** Comparison of T3 levels before and after vitamin D administration in intervention and control groups Data are expressed as mean±SD. p<0.05 indicates statistical significant difference in intervention group compared to control group after trial end. T3: triiodothyronine

Study		Intervention	Control	P-value
Chahardoli et al. 2019 [13]	Baseline (ng/mL)	1.28±0.34	1.32±0.37	0.77
	After Trial	1.28±0.35	1.31±0.34	
Jiang et al. 2023 [17]	Baseline (pmol/L)	4.64±0.72	4.66±0.82	0.000
	After Trial	4.84±0.92	4.30±0.648	
Mei et al. 2021 [18]	Baseline (pmol/L)	18.13±2.38	16.52±2.96	<0.001
	After Trial	4.55±0.71	5.10±1.01	

Six of the studies explored the changes in mean T4 levels following vitamin D supplementation, as shown in Table 6. The study by Behera et al. demonstrated a significant decrease in mean T4 levels at the end of the trial as compared to baseline levels [16]. The remaining studies, conducted in three months or less, demonstrated no significant changes in T4 levels as compared to control group following trial end [13-15]. On the other hand, studies with a longer duration of follow-up demonstrated a significant change in mean T4 levels in the intervention group at trial end compared to control group [17-18].

**Table 6:** Comparison of T4 levels before and after vitamin D administration in intervention and control groups Data are expressed as mean ± SD. p < 0.05 indicates a statistical significant difference in the intervention group compared to the control group after trial end. *statistically significant decrease as compared to baseline in the intervention group. T4: thyroxine

Study		Intervention	Control	P-value
Chahardoli et al. 2019 [13]	Baseline (μg/dL)	11.35±1.82	12.1±1.99	0.4
	After Trial	10.7±1.58	11.1±1.52	
Simsek et al. 2016 [14]	Baseline (ng/dL)	1.5±1.1	1.1±0.4	0.329
	After Trial	1.2±0.4	1.4±1.3	
Chaudhary et al. 2016 [15]	Baseline (pmol/L)	13.90±3.86	14.03±3.99	0.468
	After Trial	16.47±2.06	16.86±1.93	
Behera et al. 2020 [16]	Baseline (pmol/L)	16.08±2.79	15.91±2.50	0.000
	After Trial	17.38±2.97	15.15±1.93	
Jiang et al. 2023 [17]	Baseline (pmol/L)	54.89±8.24	47.61±8.08	<0.001
	After Trial	10.98±2.27	10.44±2.09	
Mei et al. 2021 [18]	Baseline (ng/dL)	0.9±0.29	N/A	0.005*
	After Trial	1.11±0.198	N/A	

Discussion

This section explores the changes associated with vitamin D supplementation in TSH, T3, and T4 levels. A study by Mackawy et al. concluded that patients with hypothyroidism secondary to AITD had concomitant low levels of vitamin D, and there was a negative significant correlation between vitamin D and TSH levels. Furthermore, they suggested that the degree and severity of hypothyroidism were significantly associated with serum vitamin D levels [20].

All studies included in this review with a follow-up duration of six months or more showed significant changes in TSH levels compared to those with a shorter duration. A p-value < 0.05 across all studies denoted statistical significance. These significant changes highlight the importance of long-term follow-up. This finding aligns with a meta-analysis by Zhang et al., which concluded that vitamin D supplementation reduces antibody titers in patients with Hashimoto's thyroiditis when the treatment duration exceeds three months [21]. Accordingly, the studies were categorized based on their follow-up periods into short term, which lasted three months or less, and long-term, which lasted more than three months.

Short-Term Follow-Up Period

The studies conducted by Vahabi et al. and Chahardoli et al. involved participants diagnosed with Hashimoto's thyroiditis, treated with levothyroxine, and followed up for only three months [12,13]. Similarly, in both studies, the mean serum TSH levels in the vitamin D-supplemented group showed no significant changes at the end of the study period compared to the placebo (p = 0.16 and p = 0.47, respectively) [12,13].

The study by Simsek et al. included participants with GD or HT; however, due to the low number of patients with GD, both AITDs were not compared separately in the results section [14]. The participants were randomized into an intervention group, which received 1,000 IU of vitamin D per day for one month, and a control group, which did not receive any intervention. Furthermore, symptomatic patients in both groups were initiated on either levothyroxine or methimazole therapy [14]. The asymptomatic patients were diagnosed with subclinical thyroid disease. In a parallel fashion to the previous two studies, mean TSH levels did not show a significant decrease compared to the placebo after one month of vitamin D supplementation (p = 0.265). Additionally, mean TSH levels compared to baseline did not demonstrate a significant decrease (p = 0.980) [14].

The final study included in this section was by Chaudhary et al. [15]. This was a randomized open-label trial. Newly diagnosed AITD participants were randomized into two groups: a treatment group receiving vitamin D and calcium carbonate and a control group receiving daily calcium carbonate for eight weeks. Thirty-two of the participants were receiving levothyroxine during the study period. Comparison of baseline mean TSH levels and three months post-treatment in the intervention group compared to the control group showed no significant changes (p = 0.605) [15].

The above findings are consistent with Tang et al.'s systematic review and meta-analysis, which demonstrated that improvements in thyroid function require more than 12 weeks of intervention [22].

Long-Term Follow-Up Period

The study by Behera et al. was a randomized open-label trial [16]. This was the only study that did not include a control group. Participants with thyroid autoimmunity were included, established by the presence of high thyroid auto-antibody titers. The participants were administered vitamin D weekly for two months, followed by monthly doses for the remaining four months [16]. The study found a significant reduction in the levels of mean TSH as compared to baseline, decreasing from 7.23 ± 3.16 mIU/L to 3.04 ± 2.62 mIU/L (p = 0.001) [16].

The two studies included in this review by Jiang et al. and Mei et al., both conducted in China, demonstrated significant changes in mean TSH levels compared to the control group, at the end of the trial (p = <0.001) [17,18]. Furthermore, the study conducted by Mei et al. demonstrated normalization of the TSH levels in the vitamin D group, which increased from 0.038 ± 0.031 IU/L to 1.47 ± 0.78 IU/L [18]. As a result of these findings, the authors determined that the level of thyroid function in hyperthyroidism combined with hypercalcemia can be improved by adjuvant vitamin D3 therapy [18]. These findings could be explained by a systematic review by Liu et al., which concluded that there is a low vitamin D status among the population of Mainland China, particularly among adults [23].

The study by Mei et al. served to determine the efficacy of vitamin D3 treatment in patients with newly diagnosed Graves' disease with concomitant hypercalcemia, as a secondary outcome [18]. Both intervention and control groups received methimazole (ATD) [18]. On the other hand, the study analyzed by Jiang et al included patients with established AITD, evidenced by elevated serum anti-TPO and/or anti-Tg levels [17]. The intervention group was further divided into two sub-groups, one of which received 800 IU of vitamin daily and the second, which received vitamin D and levothyroxine. In addition to the significant decrease in mean TSH in the intervention group as compared to placebo at the end of the trial (p = 0.000), both vitamin D and vitamin D/levothyroxine sub-groups revealed a significant reduction in mean TSH levels as compared to baseline: 3.35 ± 1.82 mIU/L to 2.49 ± 1.22 mIU/L (p = < 0.0001) and 3.48 ± 1.25 mIU/L to 1.99 ± 1.18 mIU/L (p = 0.0001), respectively [17].

T3 Levels

The study by Chahardoli et al. demonstrated no changes in T3 levels in both intervention and control groups versus baseline values (p = 0.99) and placebo (p = 0.77) [13]. These findings contrast with the studies by Jiang et al. and Mei et al., which showed a significant increase in free triiodothyronine (fT3) compared to placebo in the former (p = 0.000) and a significant decrease compared to placebo in the latter (p = < 0.001) [17,18]. These findings mirror the changes seen in TSH levels, in each of the respective studies.

T4 Levels

All of the studies measured the levels of free thyroxine (fT4), except for the study by Chahardoli et al., which measured T4 levels [13]. Despite this, the studies conducted in three months or less demonstrated no significant changes in mean fT4 levels following vitamin D supplementation (Chahardoli et al., p = 0.4; Simsek et al., p = 0.32; Chaudhary et al., p = 0.46) [13-15].

In contrast, the study by Jiang et al. not only found a statistically significant increase in mean fT4 levels as compared to placebo at the end of the trial (p = 0.000), but also the mean levels of fT4 increase from baseline was found to be statistically significant in both sub-group analyses [17]. The fT4 levels in the group treated with vitamin D only increased from 16.07 ± 3.42 pmol/L to 16.84 ± 3.45 pmol/L (p = 0.0157), and the group treated with both vitamin D and levothyroxine increased from 16.09±1.88 pmol/L to 17.99 ± 2.20 pmol/L (p = 0.0001) [17].

Limitations

The limitations of this systematic review include the use of only freely accessible full-text articles and the limited number of studies reviewed. This is in addition to the limitations in each of the separate studies, including small sample sizes and limited study duration. Four of the seven studies had a limited study duration, which may have contributed to the lack of significant changes in vitamin D levels.

## Conclusions

In conclusion, the effect of vitamin D on thyroid function appears to vary significantly across different studies. Only three of the included studies demonstrated significant changes in TSH levels, indicating the need for further research to establish a definitive conclusion and establish a causal link. The influence of vitamin D on triiodothyronine exhibited significant changes in two studies, while the changes in thyroxine levels were consistent with TSH. Additionally, the impact of pre-existing vitamin D deficiency in these patients requires further investigation to determine any significant association between vitamin D levels and thyroid hormone levels. Importantly, future research should prioritize longer-term studies to observe thyroid function changes following extended supplementation, as evidence indicates that the duration of supplementation plays a crucial role in observing significant changes in TSH levels. This is largely in agreement with the studies published, which also show varying responses to vitamin D supplementation.
